# Secretome analysis reveals effector candidates associated with broad host range necrotrophy in the fungal plant pathogen *Sclerotinia sclerotiorum*

**DOI:** 10.1186/1471-2164-15-336

**Published:** 2014-05-04

**Authors:** Koanna Guyon, Claudine Balagué, Dominique Roby, Sylvain Raffaele

**Affiliations:** INRA, Laboratoire des Interactions Plantes-Microorganismes (LIPM), UMR441, F-31326 Castanet-Tolosan, France; CNRS, Laboratoire des Interactions Plantes-Microorganismes (LIPM), UMR2594, F-31326 Castanet-Tolosan, France

**Keywords:** *Sclerotinia sclerotiorum.* Effectors, Gene expression, Secretome, Necrotrophic fungal, Pathogen, *Arabidopsis thaliana*

## Abstract

**Background:**

The white mold fungus *Sclerotinia sclerotiorum* is a devastating necrotrophic plant pathogen with a remarkably broad host range. The interaction of necrotrophs with their hosts is more complex than initially thought, and still poorly understood.

**Results:**

We combined bioinformatics approaches to determine the repertoire of *S. sclerotiorum* effector candidates and conducted detailed sequence and expression analyses on selected candidates. We identified 486 *S. sclerotiorum* secreted protein genes expressed *in planta*, many of which have no predicted enzymatic activity and may be involved in the interaction between the fungus and its hosts. We focused on those showing (i) protein domains and motifs found in known fungal effectors, (ii) signatures of positive selection, (iii) recent gene duplication, or (iv) being *S. sclerotiorum*-specific. We identified 78 effector candidates based on these properties. We analyzed the expression pattern of 16 representative effector candidate genes on four host plants and revealed diverse expression patterns.

**Conclusions:**

These results reveal diverse predicted functions and expression patterns in the repertoire of *S. sclerotiorum* effector candidates. They will facilitate the functional analysis of fungal pathogenicity determinants and should prove useful in the search for plant quantitative disease resistance components active against the white mold.

**Electronic supplementary material:**

The online version of this article (doi:10.1186/1471-2164-15-336) contains supplementary material, which is available to authorized users.

## Background

The white mold fungus *Sclerotinia sclerotiorum* (Lib.) de Bary is a cosmopolitan necrotrophic pathogen infecting over 400 plant species. It is among the most devastating pathogens of soybean, rapeseed and sunflower, causing several hundred million dollar losses annually at the pre- and postharvest stages [1]. *S. sclerotiorum* host range is remarkably broad, with fruit and vegetable productions also being severely impacted [2]. *S. sclerotiorum* and its close relative the grey mould fungus *Botrytis cinerea* are among the few fungal pathogens considered as typical necrotrophs. As such, they derive energy to complete their life cycle mostly from dead plant cells, as opposed to biotrophs that feed on living plant cells.

There is now ample evidence that biotrophic and hemibiotrophic fungi secrete specialized effector proteins manipulating host cell physiology to obtain nutrients, suppress plant defense and ultimately promote infection [3]. Effectors may also trigger plant defense responses, leading to plant resistance, when recognized directly or indirectly by the plant immune system in a gene-for-gene relationship. This results from a co-evolutionary arms race between pathogen effectors, their plant targets, and components of the plant immune system [4]. Necrotrophs have long been considered as less adapted, secreting mostly degrading enzymes and toxins that unspecifically trigger programmed cell death (PCD) and dismantle plant cells.

However, host specific necrotrophs such as *Cochliobolus victoriae* secrete effector proteins translocated into plant cells that interact with specific corresponding host proteins to facilitate disease progression [5, 6]. This involves the activation of plant PCD instead of its suppression as in the case of infection by biotrophic pathogens. *S. sclerotiorum* also produces the non-proteic pathogenicity determinant oxalic acid. This molecule induces the synthesis of reactive oxygen species (ROS) and triggers plant PCD late during infection, but has the opposite effect, suppressing ROS burst and PCD induction, at the early stages of infection [7]. The SSITL secreted integrin-like protein of *S. sclerotiorum* promotes virulence and delays the activation of plant defense responses, supporting the view that *S. sclerotiorum* secretes effectors to finely manipulate plant physiology [8]. In addition, enzymes secreted by necrotrophs can act as virulence factors independently of their catalytic activity [9]. Effector repertoires vary considerably, notably according to pathogens lifestyle [10], and it is becoming clear that interactions between necrotrophs and their host plants are considerably more complex and subtle than previously considered. What is the effector candidate repertoire associated with broad host range necrotrophy remains unclear. As a first step towards elucidating the molecular bases for colonization by *S. sclerotiorum*, its repertoire of effector candidates needs to be determined.

The recent release of genome sequences for a number of plant pathogenic fungi facilitated the search for effector candidates (ECs) at the genome level [11]. Nevertheless, considering that pathogen effector repertoires are typically lineage-specific, the identification of effectors remains challenging [4]. The analysis of *S. sclerotiorum* genome sequence uncovered sets of genes associated with the manipulation of redox status, including enzymes of OA biosynthesis, the degradation of plant cell wall, and 603 secreted proteins with other functions [12]. Known hallmarks of fungal effectors such as the presence of signal peptides and absence of transmembrane domains, small size and amino-acid composition generally produce lists of hundreds of potential effectors in a given pathogen. Therefore, more sophisticated approaches are required to pinpoint the most relevant ECs for the promotion of infection in *S. sclerotiorum* secretome.

A limited number of known fungal effector families show conservation at the sequence level or similar predicted functions. This is notably the case for the toxin and cell death elicitor proteins of the Necrosis and ethylene-inducing Like Proteins (NLPs), the cerato-platanin, cyanovirin-N homology (CVNH) and ECP2 families [13–16]. The growing number of characterized fungal effectors suggests conservation at the biochemical function level in the overall effector repertoire of several fungal pathogens. The ability to bind chitin or other cell wall oligosaccharides, masking the presence of the pathogen or dampening damage-induced plant responses, is a feature common to effectors from multiple fungal pathogens [17–20]. Fungal effectors harboring a protease inhibitor activity are also common [21–24]. The biochemical activity of a few other fungal effectors such as *M. oryzae* Fungalysin metalloprotease AvrPita [25], *U. maydis* chorismate mutase cmu1 [26] and peroxidase inhibitor PEP1 [27] may also be part of the arsenal of effector functions in multiple fungal lineages. This hypothesis suggests that thorough annotation of protein domains and prediction of biochemical function of secreted proteins may prove useful to identify novel effectors in *S. sclerotiorum*.

However, a majority of effectors do not show significant similarity to known sequences in other organisms nor obvious protein domains. Yet other genomic characteristics may help identify EC genes. The rapid evolution of effector genes allows the fungi to overcome selection pressures induced by resistant plant cultivars. A high ratio of non-synonymous over synonymous substitutions (Ka/Ks) in alleles from related strains is a frequently used proxy for inferring fast gene evolution and the action of positive selection [28]. This approach has been used to reveal ECs in several filamentous plant pathogen lineages [29–34]. Positive selection has been detected in *B. cinerea* genome [35] suggesting that it may be used to mine *S. sclerotiorum* genome for ECs. Second, gene duplication is another hallmark of several known fungal effector genes, such as the ToxB host specific toxin of *Pyrenophora tritici-repentis* [36, 37]. Third, genomic regions with high repeat and transposable element content are enriched in effector genes in several lineages of plant pathogens [37–39] suggesting that genome architecture analysis can assist in the search for EC genes. Finally, effectors can alter host cell function by mimicking plant peptides [40]. These ECs likely elude functional annotation on the basis of primary amino-acid sequence, but may be revealed using three-dimensional structure prediction.

As opposed to Oomycete pathogen genomes in which many effector genes can be identified through conserved sequence motifs [29, 41], the use of conserved sequence motifs, such as the Y/F/WxC motif [42], has proven limited in revealing fungal effectors across lineages. However, the presence of a signal peptide directing protein secretion and gene expression *in planta* are relatively universal properties of effectors that can be exploited as first filters to narrow down the list of effector candidates in fungal genomes.

*S. sclerotiorum* effector proteins would be useful as probes to search for resistance components in plants and to design strategies for inhibiting infections by this devastating but poorly characterized pathogen. In this study, we report a diverse repertoire of *S. sclerotiorum* effector candidates revealed by an in depth analysis of its predicted secretome. We combined refined secretome annotation, phylogeny, selection and gene duplication analyses, and three-dimensional structure prediction to identify 78 ECs. Among those, we highlight a predicted subtilisin inhibitor, a xylanase, a duplicated gene of unknown function and three toxin mimics as high priority candidates for functional studies. We analyzed *in planta* expression pattern for 16 EC genes and revealed host-blind and host-regulated ECs.

## Results

### Definition and annotation of ***S. sclerotiorum*** secretome

**Figure 1:**
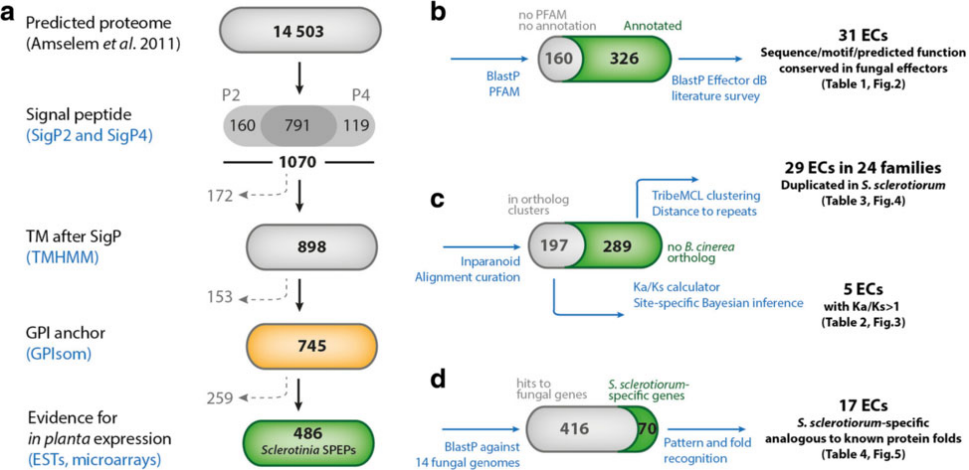
***S*** . ***sclerotiorum*** **secretome prediction and analysis pipeline. (a)** secretome analysis pipeline. We identified 745 predicted secreted proteins (yellow box) among which 486 showing experimental evidence for expression *in planta* (*S. sclerotiorum* *s*ecreted *p*roteins *e*xpressed *in* *p* *lanta*, SPEPs). The number of proteins filtered out is indicated in grey with dotted arrows, the number of selected proteins is given within boxes, bioinformatics tools and resources used are indicated in blue. **(b)** Identification of effector candidates (ECs) based on sequence, motifs or protein domains conserved in fungal effectors. **(c)** Identification of ECs belonging to duplicated gene families and showing signatures of positive selection. **(d)** Identification of *S. sclerotiorum*-specific ECs of unknown function analogous to known protein folds. The results of analyses **b**, **c** and **d** are reported in tables and figures as indicated.

In the original analysis of *S. sclerotiorum* genome, secreted proteins were predicted using SignalP, TargetP and TMHMM, and annotated using Interproscan [12]. To identify and explore candidate effectors in the genome of *S. sclerotiorum*, we built a bioinformatics workflow exploiting genomic features typical of known filamentous plant pathogen effectors. We first refined the prediction of secreted proteins combining predictions from SignalP2 and 4 to identify 1070 proteins with a secretion signal. To evaluate the sensitivity of this prediction, we applied the same methodology on a list of 1985 eukaryotic secreted proteins validated experimentally from the SPdb [43]. We retrieved 1971 proteins predicted as secreted, corresponding to a true positive rate of 99.34%. Next, we removed 172 proteins predicted by TMHMM to harbor transmembrane helices, then 153 proteins predicted by GPIsom to harbor glycophosphatidylinositol anchor motifs, that likely represent surface proteins rather than secreted effectors. This resulted in a list of 745 predicted secreted proteins. To increase the likelihood of identifying genes relevant for infection, we selected those for which there is evidence for expression *in planta* based on publicly available EST and microarrays data. We considered genes expressed, even if not induced, during interaction with rapeseed, sunflower or tomato (see Methods). Although this filtering pipeline likely excluded relevant effector candidates, it resulted in a total of 486 genes encoding predicted secreted proteins expressed *in planta* (SPEPs, Figure 1a, Additional file 1: Table S1).

Next, we performed three different effector-oriented analyses on the 486 SPEP genes. First, we used Blast2GO, PFAM domain and nuclear localization signal (NLS) searches to annotate 326 SPEPs (Figure 1b). We built a database of known fungal effectors and explored the literature to select 31 *S. sclerotiorum* ECs among annotated SPEP genes. Second, we defined clusters of orthologous genes (COGs) between *S. sclerotiorum* and *B. cinerea* predicted genes using Inparanoid (Figure 1c). A total of 197 SPEP genes grouped in COGs. We aligned *S. sclerotiorum* and *B. cinerea* orthologs for these 197 gene pairs and calculated ratios of non synonymous over synonymous substitutions (Ka/Ks) to identify five ECs with signature of positive selection. The 289 SPEP genes with no ortholog in *B. cinerea* were grouped into clusters based on sequence similarity to identify 29 ECs distributed in 24 families containing genes duplicated in *S. sclerotiorum*. Finally, we analyzed the taxonomic distribution of SPEP genes across the kingdom Fungi using BlastP searches against a database of 14 complete genomes representative of all major fungal lineages. This identified 70 *S. sclerotiorum*-specific SPEP genes, most of which had no annotation (Figure 1d). We used protein structure prediction and pattern and fold recognition searches to identify 17 ECs analogous to known protein fold encoded by *S. sclerotiorum*-specific SPEP genes.

Using effector-oriented analyses, we identified four lists of ECs, containing a total of 78 EC genes (four being common to two lists). We could not predict any enzymatic activity encoded by 33% of the SPEP genes (160), suggesting that *S. sclerotiorum* effector repertoire encodes diverse functions that are not restricted to plant cell degrading enzymes. Besides, 59.5% of the SPEP genes (289) did not cluster in *B. cinerea* COGs, revealing a relatively high degree of divergence from this closely related fungal pathogen.

### *Sclerotinia* effector candidates showing conserved domains

Some fungal effectors show conserved protein domains and biochemical functions that can be identified at the protein sequence level. In a first approach to identify ECs, we used (i) PFAM annotations, (ii) nuclear localization signal (NLS) predictions, and (iii) BlastP searches against known fungal effectors. We found 326 annotated SPEP genes among which we selected 13 as effector candidates based on the presence of specific PFAM domains (Table 1). Indeed, six predicted SPEPs (SS1G_00642, SS1G_02014, SS1G_04786, SS1G_09392, SS1G_12336, SS1G_14184) included chitin-recognition or chitin-binding domains (PF00187, PF03067) and one SPEP (SS1G_12509) contained LysM domains (PF01476). SS1G_07836 was annotated as a concanavalin A-like lectin/glucanase that binds to complex carbohydrates, and harbors a Peptidase A4 (PF01828) with a typical lectin fold. SS1G_08698 contains a Ricin type beta-trefoil lectin domain (PF00652) typical of ricin-like toxins. These predicted SPEPs are relevant ECs considering that chitin- and carbohydrate-binding activity has been reported for several fungal effector families. We also selected three predicted SPEPs (SS1G_01593, SS1G_03282, SS1G_12605) with protease inhibitor domains (PF05922), another activity found in known fungal effectors. We identified two predicted SPEPs (SS1G_03611 and SS1G_13935) with a CFEM cystein-rich fungal effector motif (PF05730). Second, we selected another 11 SPEP genes based on the presence of at least one predicted NLS in their sequence (Table 1). The co-occurrence of a secretion signal and a NLS in encoded proteins suggest that they may be active in the plant nucleus. Consistent with a nuclear activity, SS1G_01866 and SS1G_05895 also harbored transcription factor domains. Third, we selected 7 predicted SPEPs that were retrieved based on sequence similarity to known effectors or ECs from other plant pathogenic fungi (Table 1). We recovered the two necrosis and ethylene inducing peptides SsNEP1 and SsNEP2 (SS1G_03080 and SS1G_11912) described in [44] sharing ~40% identity with various NEP-like proteins, and harboring a characteristic PFAM domain (PF05630). We selected SS1G_00849 for sharing 59% identity with *Colletotrichum hingginsianum* effector candidate 91 (CHEC91, [45]). There was no protein domain identified in this protein but three-dimensional structure prediction indicates that it is analogous to *Alternaria alternata* AltA-1 allergen. We selected SS1G_02904 for sharing 35% identity with *C. hingginsianum* effector candidate 80 (CHEC80, [45]). It features a Cyanovirin-N homology domain (PF13639) that corresponds to a carbohydrate-binding module [15, 46]. We identified SS1G_08858 showing 25% identity with *M. oryzae* effector AvrPita (MGG_15730, [25]) and harboring a M35 metalloprotease domain (PF02102). Finally we selected SS1G_10096 as similar (>60% identity) to pathogen-associated molecular patterns (PAMP) with a cerato-platanin domain (PF07249) [14, 45, 47, 48]. These 31 ECs identified based on protein annotations showed that the activity of *S. sclerotiorum* secretome may not be limited to typical cell death elicitors but is predicted to cover a wide range of functions known for fungal effectors, including chitin binding, proteases and protease inhibitors.

**Table 1:** **List of 31** ***S*** . ***sclerotiorum*** **effector candidates selected based on their annotation**

SPEPs containing PFAM domains found in fungal effectors				
Protein ID	Annotation	PFAM domain(s)	Length	Comments
SS1G_00642	Chitin binding protein	Chitin recognition protein (PF00187.14)	563	
SS1G_02014	Chitin binding protein	Chitin binding domain (PF03067)	426	
SS1G_04786	Chitin binding protein	Chitin recognition protein (PF00187.14)	399	
SS1G_09392	Starch binding domain containing protein	Chitin binding domain (PF03067)	398	
SS1G_12336	Chitin binding protein	Chitin binding domain (PF03067)	294	
SS1G_14184	Agglutinin isolectin 3-like	Chitin recognition protein (PF00187.14)	245	
SS1G_12509	LysM domain protein	LysM (PF01476)	447	
SS1G_07836	Concanavalin A lectin glucanase	Peptidase_A4 (PF01828)	252	
SS1G_08698	Ricin-type toxin	Ricin-type beta-trefoil lectin domain (PF00652)	409	
SS1G_01593	Serine protease inhibitor	Peptidase inhibitor I9 (PF05922)	95	
SS1G_03282	Serine protease inhibitor	Peptidase inhibitor I9 (PF05922)	522	
SS1G_12605	Alkaline serine protease alp1	Peptidase inhibitor I9 (PF05922)	400	
SS1G_03611	Cystein-rich protein	CFEM (PF05730)	119	
SS1G_13935	Cystein-rich protein	CFEM (PF05730)	529	
**SPEPs containing nuclear localization signal (NLS)**				
**Protein ID**	**Annotation**	**PFAM domain(s)**	**Length**	**Comments**
SS1G_01866	Ring-7 protein	PHD-like zinc-binding domain (PF13771); Ring finger domain (PF13639)	425	NLS (118–141)
SS1G_03146	-	-	193	NLS (147–155)
SS1G_04309	-	-	177	NLS (82–87)
SS1G_05895	Zinc finger CCCH-type domain containing protein	Zinc finger C-x8-C-x5-C-x3-H type (PF00642)	310	NLS (256–291)
SS1G_05938	-	-	212	NLS (143–150)
SS1G_06787	-	-	430	NLS (189–250; 340–349)
SS1G_06890	-	-	284	NLS (66–120; 143–167; 225–245)
SS1G_07404	-	DUF3108 (PF11306)	282	NLS (65–79; 118–138; 223–233)
SS1G_09050	-	-	454	NLS (410–413)
SS1G_11108	Ribosomal protein s17	-	373	NLS (338–348)
SS1G_13142	-	-	131	NLS (89–99)
**SPEPs showing homology to fungal effectors or fungal effector candidates**				
**Protein ID**	**Annotation**	**PFAM domain(s)**	**Length**	**Comments**
SS1G_03080	SsNEP1	Necrosis inducing protein (PF05630)	246	Homolog to *B. cinerea* NEP1 (Staats et al., 2007); P. Sojae NIP (Qutob et al., 2002); studied in (Bashi et al., 2011)
SS1G_11912	SsNEP2	Necrosis inducing protein (PF05630)	245	Homolog to *B. cinerea* NEP1 (Staats et al., 2007); P. Sojae NIP (Qutob et al., 2002); studied in (Bashi et al., 2011)
SS1G_00849	AltA-1 allergen analog	-	152	Homolog to *C. hingginsianum* HE651255_CHEC91 Kleemann et al., 2012)
SS1G_02904	CVNH protein	CyanoVirin-N Homology domain (PF08881)	169	Homolog to *C. hingginsianum* HE651243_CHEC80 Kleemann et al., 2012)
SS1G_08858	Deuterolysin metalloprotease	Deuterolysin metalloprotease (M35) family (PF02102)	354	Homolog to *M. oryzae* AvrPita (MGG_15370 - Orbach et al., 2000)
SS1G_10096		Cerato-platanin (PF07249)	137	Homolog to *B. cinerea* BcSpl1 (Frias et al., 2011); *H. atroviridis* EPL1 (Seidl et al., 2006); *M. oryzae* MSP1 Jeong et al., 2007); *C. hinginsianum* HE651160_CHEC5 Kleemann et al., 2012)

Results from Blast2GO automated annotation, PFAM domain searches and BlastP against known fungal effectors were considered. Table entries are ordered as in main text. SPEP, *S. sclerotiorum* secreted protein induced *in planta*.

### A family of subtilisin-inhibitor effector candidates conserved in Ascomycetes

**Figure 2:**
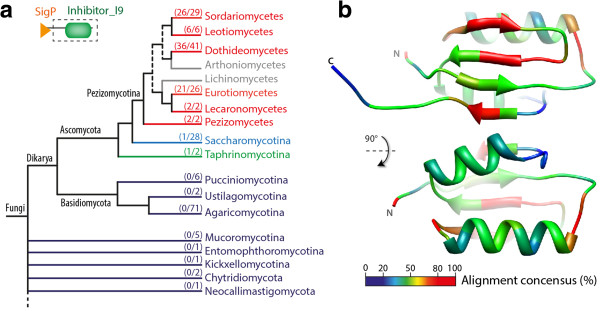
***Sclerotinia*** **effector candidates showing conserved domains: example of a novel class of protease inhibitors. (a)** The domain organization of SS1G_01593, a *S. sclerotiorum* effector candidate with a peptidase inhibitor I9 domain (PF05922), and the distribution of its homologs across fungal taxonomy shown on a tree assembled based on published phylogenies, with branches color-coded from blue to red based on the percentage of species in a given order harboring homologs. **(b)** The predicted 3D structure of SS1G_01593 with residues color-coded based on conservation in fungi.

Effectors with a protease inhibitor activity have been described in fungal plant pathogens with a biotrophic phase of infection [22, 49]. We identified three *S. sclerotiorum* candidate effectors (SS1G_01593, SS1G_03282 and SS1G_12605, Table 1) with a serine protease inhibitor I9 domain (PF05922) that corresponds to the propeptide inhibitor domain of subtilisins. To document the taxonomic distribution of SS1G_01593 homologs in fungi, we performed a BlastP search against the predicted proteome of 234 fungal species. Using signal peptide predictions, we identified 99 secreted homologs across 97 species. Secreted homologs of SS1G_01593 are restricted to Ascomycetes and found in all Leotiomycete species considered (Figure 2a). At the sequence level, homologs of SS1G_01593 are clearly distinct from *C. fulvum* Avr2*, U. maydis* Pit2, *Uromyces fabae* RTP1p and *M. lini* AvrP123, suggesting that SS1G_01593 family represent a distinct class of protease inhibitor effectors. To get insights into SS1G_01593 function and evolution, we predicted the 3D structure of the protein and mapped residue conservation among the 99 secreted homologs on this structure. SS1G_01593 protein is predicted to adopt the structure of subtilisin pro-domain consisting of 4 beta-sheets facing two alpha-helices (Figure 2b). The beta-sheets, interacting with the catalytic domain in subtilisins, are well conserved in fungi, whereas the exposed alpha-helices are more variable, suggesting that the ability to interact with subtilisins is conserved.

### *Sclerotinia* effector candidates showing high Ka/Ks ratios

**Figure 3:**
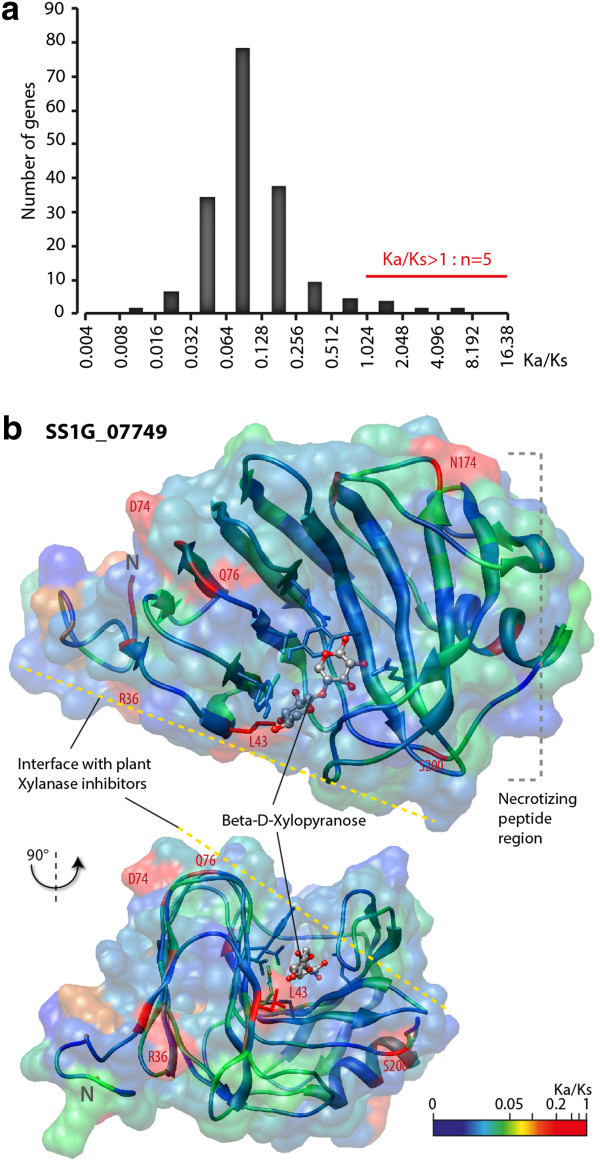
***Sclerotinia*** **effector candidates selected based on Ka/Ks ratio. (a)** Distribution of Ka/Ks ratio for the 197 SPEP genes with orthologs in *B. cinerea*, calculated with Yn00 method on pairwise ortholog alignments. **(b)** Predicted 3D protein structure of SS1G_07749, a member of the glycoside hydrolase 11 xylanase family with global Ka/Ks = 2 in comparisons with *B. cinerea* orthologs. Residues of the 3D model are color-coded according to site-specific Ka/Ks ratios calculated using Bayesian inference with M8 model [28]. Residues with Ka/Ks > 1 are labeled on the structure. A putative Beta-D-Xylopyranose substrate molecule present in the 3b5l_B model, best structural analog of SS1G_07749, is shown as balls and sticks. The side chains of residues forming the predicted substrate binding site predicted by COFACTOR are show as sticks. The interface with plant xylanase inhibitors shown as a yellow dotted line was inferred from [53, 54] and the necrotizing peptide region shown as a grey dotted line was inferred from [9].

According to the red queen hypothesis, fungal pathogen effectors are predicted to evolve rapidly. Signatures of natural selection were used to identify effectors and elicitors of immune responses in bacterial, oomycete and fungal plant pathogens [29, 35, 50, 51]. To detect signatures of natural selection in SPEP genes, we calculated Ka/Ks ratios using Yn00 [28] on alignments between *S. sclerotiorum* and *B. cinerea* 197 core ortholog pairs. We obtained Ka/Ks values ranging from 0.009 to 6, with a median value of ~0.1. Notably 5 genes (2.5%) showed Ka/Ks > 1, suggesting positive selection (Figure 3a, Table 2). To identify codon sites under positive selection in the five SPEP genes showing Ka/Ks > 1, we used a Bayesian inference approach on alignments of *S. sclerotiorum* 1980, *B. cinerea* B05.10 and *B.cinerea* t4 orthologs. We could not detect sites with Ka/Ks > 1 in SS1G_04551 and SS1G_07158 using this dataset. We detected 7, 50 and 20 sites with Ka/Ks > 1 in SS1G_07749, SS1G_10165 and SS1G_10617 mature proteins respectively. The p-value for positive selection using M8 and M8e models was 8.2e^-2^, 3.8e^-4^ and 3.0e^-1^ for SS1G_07749, SS1G_10165 and SS1G_10617 respectively (Table 2). Interestingly, *SS1G_07749* encodes a putative xylanase that may function as a virulence factor such as xylanases from other fungi [9, 52]. To get insights on the constraints shaping the evolution of SS1G_07749 xylanase, we predicted the 3D structure of SS1G_07749 mature protein and mapped the local Ka/Ks ratios on this structure (Figure 3b). All seven selected sites correspond to surface-exposed residues with three residing at the surface potentially interacting with plant xylanase inhibitors (R36, D74, Q76), three at the surface of the region corresponding to *B. cinerea* Xyn11 necrotizing peptide (N174, L189, S200) and one (L43) modifying the surface of the substrate-binding pocket. These findings are consistent with adaptation to host and the possible involvement of SS1G_07749 in virulence.

**Table 2:** **List of five** ***S*** . ***sclerotiorum*** **effector candidates selected based on Ka/Ks > 1 in pairwise comparisons with their** ***B*** . ***cinerea*** **orthologs**

Protein ID	Annotation	PFAM domains	Length	Ka/Ks vs BC1T	KaKs vs BcT4	Ka/Ks > 1 sites	Prob
SS1G_04551	Pectinesterase A	Pectinesterase (PF01095.14)	308	2	2	max Ka/Ks = 0.93	NA
SS1G_07158	-	DUF1374 (PF07118)	328	6	ND	max Ka/Ks = 0.4	NA
SS1G_07749	Xylanase	Glycoside hydrolase family 11 (PF00457.12)	200	4	4	R36, L43, D74, Q76, S128, N174, S200	0.08224
SS1G_10165	Pectinesterase	Pectinesterase (PF01095.14)	310	2	0,061669	P22, K31, T34, A36, S54, A63, S85, S86, G88, S89, Q95, A118, D146, I165, F191, D208, P211, S212, T213, L218, 226I, 230A, 233S, 236A, 237G, 238 T, S246, V253, M258, S259, N260, 261 V, N263, V269, S274, P275, N276, 278Q, H285, A286, A290, H301, S302, P306, S310, N316, K318, S319, S324	0.00038
SS1G_10617	Glycoside hydrolase family 15 protein	Glycoside hydrolase family 15 (PF00723.16); Carbohydrate-binding module family 20 (PF00686.14); Carbohydrate-binding module family 25 (PF03423.8)	628	2	0,078172	N53, R56, M81, S91, N128, S313, S361, Q383, Q409, S416, N514, Y526, F537, V549, K586, V589, S605, S617, Q639	0.2987

Site specific Ka/Ks ratio estimated using Bayesian inference based on the M8 model of Yang *et al*., 2000. Only sites with Ka/Ks > 1 in the mature protein sequence are reported. P-value for positive selection estimated using a likelihood ratio test based on the comparison of twice the log likelihood difference obtained with the M8 and M8a null model with a chi-square probability table of degree of freedom 1. BC1T, *Botrytis cinerea* B.05 genome; BcT4, *Botrytis cinerea* T4 genome; ND, not determined.

### *Sclerotinia* effector candidates encoded by recently duplicated genes

**Figure 4:**
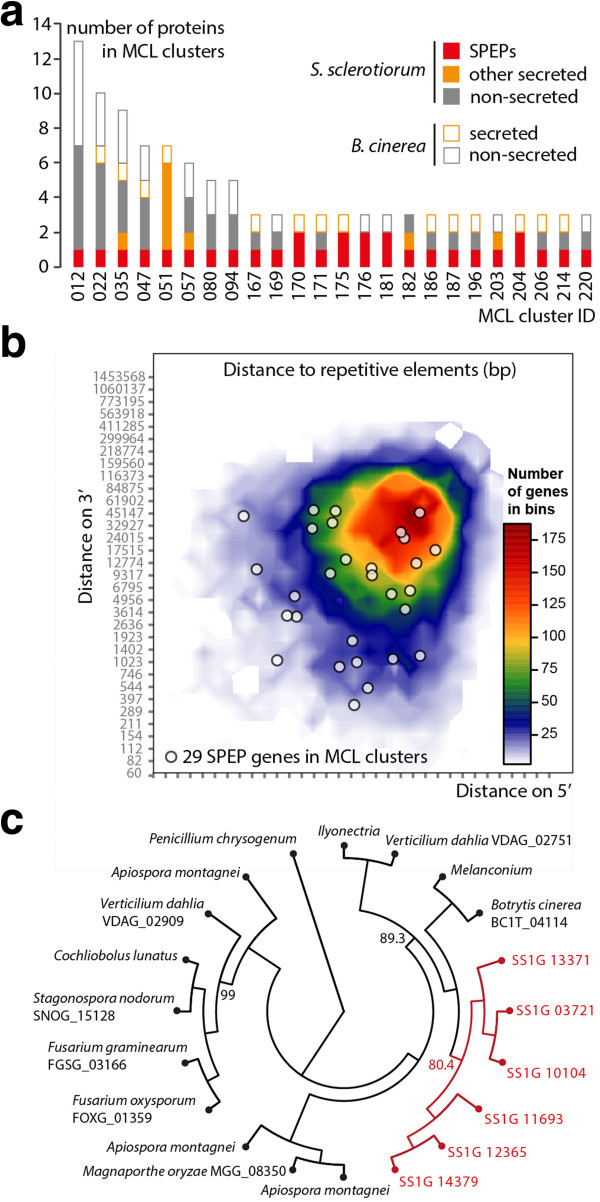
**Expansion of effector candidate families in** ***S*** . ***sclerotiorum*** **genome. (a)** Composition of clusters determined by Markov clustering of *S. sclerotiorum* and *B. cinerea* complete proteomes (MCL clusters) containing putative duplicated *S. sclerotiorum* SPEP genes. **(b)** Distribution of distances to the closest repetitive genomic element on 5’ and 3’ side for all genes (heatmap) and the genes encoding the 29 SPEPs in MCL clusters (dots). **(c)** Parsimony phylogenetic tree of SS1G_13371 SPEP and its 19 closest homologs. Bootstrap support calculated over 100 replicates is shown for the major branches, *S. sclerotiorum* clade is shown in red.

Several effectors of filamentous plant pathogens evolved through gene duplication followed by rapid diversification [16, 51]. To identify SPEP genes that underwent duplications since the divergence of *S. sclerotiorum* with *B. cinerea,* we grouped proteins from their whole predicted proteomes based on sequence similarity using Markov clustering, and selected groups matching all three criterions: (i) containing at least 3 proteins, (ii) containing at least one *S. sclerotiorum* SPEP gene and (iii) containing more than 50% of proteins from *S. sclerotiorum*. We obtained 24 such groups including a total of 71 *S. sclerotiorum* proteins among which 29 *S. sclerotiorum* SPEP genes (Figure 4a, Table 3). Groups 051, 170, 175 and 204 contained only secreted proteins and probably carry effector-specific functions. Group 182 and 167 contained SPEP genes identified as ECs based on the presence of CFEM and LysM domains respectively (Table 1). SPEP genes from group 51, 80 and 204 had no conserved domain or homology to proteins of known function. In some pathogen genomes, the expansion of effector gene families is associated with the proximity to transposable elements [4]. To determine the repeat environment of *S. sclerotiorum* ECs, we annotated repeats in *S. sclerotiorum* genome, and calculated for all genes the distance to the nearest repeat. Median distance to repeats is 7.89 Kbp across *S. sclerotiorum* genome and 3.56 Kbp for the 29 ECs that underwent recent duplications. Nine duplicated ECs are located less than 2Kbp apart from a retrotransposon and may have undergone duplication due to transposable element activity (Figure 4b, Table 3).

**Table 3:** **The 24 gene clusters containing duplicated** ***S*** . ***sclerotiorum*** **SPEP genes**

Group	N° Ss - Bc*	***S. sclerotiorum SPEPs***	Other genes in group	SPEPs annotation	Nearest repeat to SPEPs (distance Kb)
Cluster012	7-6	SS1G_01081	BC1G_01968, SS1G_09509, BC1G_01095, SS1G_00547, BC1G_09386, SS1G_09141, SS1G_05689, BC1G_02407, SS1G_06186, BC1G_12856, SS1G_02784, BC1G_13021	Catalase	5SrRNA_AN (41.6)
Cluster022	6-4	SS1G_04468	SS1G_04513, SS1G_09104, SS1G_09671, BC1G_12617, BC1G_09286, SS1G_09338, BC1G_00394, SS1G_14236, BC1G_00455	Glycoside hydrolase family 47 protein	Helitron-2_PSt (47.3)
Cluster035	5-4	SS1G_10949	BC1G_01945, SS1G_12508, SS1G_12939, BC1G_02687, BC1G_11888, BC1G_10788, SS1G_01984, SS1G_14293	Glucose oxidase	BOTY_LTR (0.8)
Cluster047	4-3	SS1G_11700	SS1G_08020, BC1G_05350, BC1G_01594, SS1G_11304, BC1G_11407, SS1G_05897	Glycoside hydrolase family 18 protein	BOTY_LTR (1.0)
Cluster051	6-1	SS1G_13371	BC1G_04114, SS1G_12365, SS1G_03721, SS1G_14379, SS1G_11693, SS1G_10104	-	Tad1-14_BG (1.1)
Cluster057	4-2	SS1G_05454	BC1G_01964, SS1G_12510, SS1G_00677, SS1G_00773, BC1G_00533	Glycosyl hydrolases family 18 protein	BOTY_LTR (0.9)
Cluster080	3-2	SS1G_05073	BC1G_10397, SS1G_10773, BC1G_07160, SS1G_13589	-	BOTY_LTR (5.8)
Cluster094	3-2	SS1G_09630	SS1G_03681, SS1G_10564, BC1G_10623, BC1G_03527	Pyrroline-5-carboxylate reductase	BOTY_LTR (3.6)
Cluster167	2-1	SS1G_13935	BC1G_12793, SS1G_13934	CFEM domain containing	BOTY_LTR (9.6)
Cluster169	2-1	SS1G_02369	SS1G_00501, BC1G_00594	Xyloglucan-specific endo-betaglucanase A	BOTY_LTR (0.5)
Cluster170	2-1	SS1G_04264, SS1G_12024	BC1G_15278	Cell wall glucanase	BOTY_LTR (13.0) – Gypsy-31_ADe-I ( 35.9)
Cluster171	2-1	SS1G_13501	BC1G_06328, SS1G_03093	Bacterial alpha-L-rhamnosidase domain protein	Harbinger-5_PSt (1.8)
Cluster175	2-1	SS1G_10092, SS1G_03618	BC1G_03590	Endo-beta-xylanase	BOTY_LTR (3.9) - BOTY_LTR (11.4)
Cluster176	2-1	SS1G_04958, SS1G_09225	BC1G_01026	Tripeptidyl peptidase sed3	BOTY_LTR (1.1) - Mariner-3_AF (5.9)
Cluster181	2-1	SS1G_07498, SS1G_01811	BC1G_06353	Glucose-methanol-choline oxidoreductase	BOTY_LTR (18.2) - BOTY_LTR (0.6)
Cluster182	3-1	SS1G_12509	SS1G_00772, SS1G_05453	LysM domain protein	Mariner-3_AF (8.1)
Cluster186	2-1	SS1G_04200	BC1G_00245, SS1G_01334	Alpha-mannosidase family protein	BOTY_LTR (2.2)
Cluster187	2-1	SS1G_04207	BC1G_00240, SS1G_04205	Polygalacturonase	BOTY_LTR (3.4)
Cluster196	2-1	SS1G_05273	SS1G_11068, BC1G_09997	Amidase family protein	BOTY_LTR (28.8)
Cluster203	2-1	SS1G_03160	SS1G_00233, BC1G_07555	Autophagy related lipase	BOTY_LTR (1.2)
Cluster204	2-1	SS1G_08110, SS1G_12361	BC1G_12374	-	5SrRNA_AN (2.3) - Tad1-14_BG (1.8)
Cluster206	2-1	SS1G_08104	SS1G_09795, BC1G_12379	Acetyl xylan esterase	5SrRNA_AN (6.2)
Cluster214	2-1	SS1G_00446	BC1G_00660, SS1G_05782	Heterokaryon incompatibility Het-c domain protein	SINE3-2_AO (5.4)
Cluster220	2-1	SS1G_05461	BC1G_06146, SS1G_13907	Thioesterase-like domain protein	TGATGAA)n (0.3)

Repbase nomenclature for transposons used here: BOTY_LTR, *Botrytis cinerea* gypsy-type retrotransposon; Gypsy-31_ADe-I, internal portion of retrotransposon GYPSY31; Harbinger-5_PSt, Harbinger-type DNA transposon; Mariner-3_AF, Mariner DNA transposons; SINE3-2_AO, SINE3 nonautonomous non-LTR retrotransposon; Tad1-14_BG, Tad1 Non-LTR retrotransposon from barley powdery mildew. Retrotransposons are indicated in bold. * Number of genes from *S. sclerotiorum - B. cinerea*.

Cluster051 is remarkable for containing only genes coding for secreted proteins, with only one from *B. cinerea* and 6 from *S. sclerotiorum*, such as the SPEP gene *SS1G_13371* (Table 3). To get support for the duplication of *SS1G_13371* gene ancestor in *S. sclerotiorum* lineage, we constructed a phylogenetic tree of SS1G_13371 homologs. A total of 58 homologs in 25 fungal species could be retrieved from the JGI database covering 238 complete fungal genomes. We selected the 20 closest homologs to build a parsimony tree based on a 90 amino-acid alignment (Figure 4c). For the 6 *S. sclerotiorum* genes of cluster 051, the phylogeny revealed clustering based on paralogy rather than orthology, suggesting that this family expanded after the separation of the 25 species analyzed. Gene duplication in this family may have allowed increased accumulation of the corresponding protein, neo-functionalization in some paralogs, or differential regulation.

### *S. sclerotiorum*-specific effector candidates with toxin structural analogs

**Figure 5:**
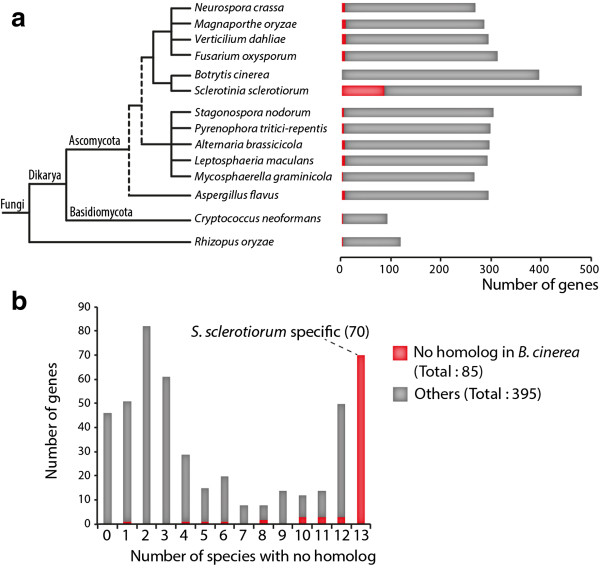
**Taxonomic distribution of** ***S*** . ***sclerotiorum*** **SPEP genes across 14 fully sequenced fungal pathogen genomes. (a)** Bar chart showing the number of *S. sclerotiorum* SPEP genes conserved along a phylogeny of fungal pathogens. Conservation was determined based on BlastP searches as described in the methods. **(b)** Distribution of *S. sclerotiorum* SPEP genes according to the number of species in which they are not conserved. *S. sclerotiorum* SPEP genes conserved in a given species but not in *B. cinerea* are shown in red.

The emergence of virulence is frequently associated with a high rate of mutation, gene gain and gene loss in effector genes. This evolutionary pattern results in a discontinuous taxonomic distribution for effector genes [4, 55]. To identify genes showing a discontinuous taxonomic distribution among *S. sclerotiorum* SPEP genes, we looked for homologs of all 486 *S. sclerotiorum* SPEP genes in the complete genome of 13 fungal pathogens with a necrotrophic phase of infection covering all major fungal lineages. As expected, the number of SPEP homologs identified in a given genome decreased with phylogenetic distance to *S. sclerotiorum*, with 395 SPEPs (81.3%) having homologs in *B. cinerea* and only 90 (18.5%) having homologs in *Cryptococcus neoformans* (Figure 5a). Forty-six SPEP genes had homologs in all 14 fungal species monitored, and 70 were *S. sclerotiorum*-specific, absent in the 13 other fungal species considered (Figure 5b). Among those, only six (8.6%) had weak matches to PFAM domains (0.004 < e-value < 0.74) other than domains of unknown function. We could not obtain information about the function of the 64 remaining SPEP genes based on annotation or homology.

**Figure 6:**
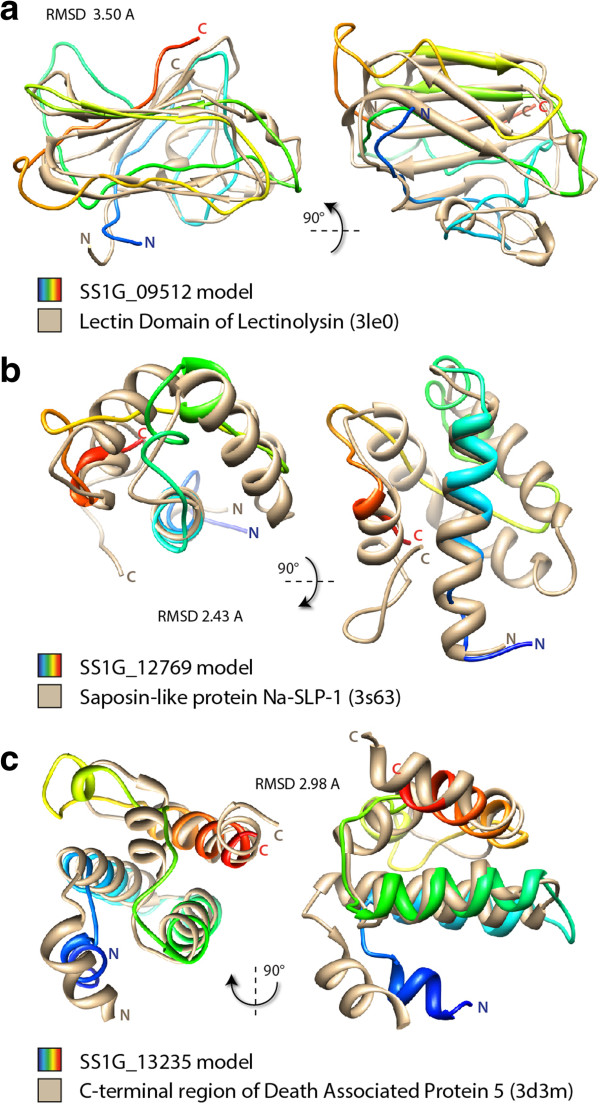
**Three** ***S*** . ***sclerotiorum*** **-specific effector candidates identified by pattern and fold recognition. (a)** Superimposed 3D protein structure of SS1G_09512 model (rainbow) and the lectin domain of *Streptococcus mitis* lectinolysin (tan). RMSD calculated by TM-align was 3.50 Å. **(b)** Superimposed 3D protein structure of SS1G_12769 model (rainbow) and *Necator americanus* Saposin-like protein Na-SLP-1 (tan). RMSD calculated by TM-align was 2.43 Å. **(c)** Superimposed 3D protein structure of SS1G_13235 model (rainbow) and the C-terminal domain of *Homo sapiens* Death Associated Protein 5 (tan). RMSD calculated by TM-align was 2.98 Å. RMSD, root mean square deviation.

To get insights into the putative function of *S. sclerotiorum*-specific predicted SPEPs with no annotation, we performed 3D protein structure predictions followed by fold-recognition searches with an aim to identify structural analogs of known function. We focused on 17 SPEP genes encoding mature proteins of at least 50 amino-acids and for which the best BlastP hit is not a *B. cinerea* protein (Table 4). We closely examined the predicted structures of three SPEPs in comparison with their structural analogs. SS1G_09512 had analogy to the lectin domain of lectinolysin, a toxin forming pores in cell membranes regulated by sugar-binding through a lectin domain [56] (Figure 6a). SS1G_12769 had analogy to the hookworm saposin-like protein Na-SLP-1, a toxin forming pores in membranes through lipid-binding activity [57] (Figure 6b). SS1G_13235 has analogy to the C-terminal domain of death-associated protein 5 (DAP5), a member of the eukaryotic translation initiation factor eIF4G protein family (Figure 6c). Cleavage of DAP5 by caspases at its C-terminus induces apoptosis [58]. SS1G_07354 also had eIF4G protein as closest analog (Table 4). We propose that these EC are toxin analogs that may have emerged through convergent evolution and could contribute to *S. sclerotiorum* virulence.

**Table 4:** **List of 17** ***Sclerotinia*** **-specific effector candidates revealed by pattern and fold recognition searches**

	Length	C-score	TM score	Analog model	Selected analog description
SS1G_00780	115	-3.14	0.610	2PFV	*S. cerevisiae* Exo70 with additional residues to 2.1 Angstrom resolution
SS1G_01817	116	-2.53	0.503	2R5M	Crystal Structure of the two MBT repeats from Sex-Comb on Midleg (SCM) in complex with peptide R-(me)K-S
SS1G_03830	155	-4.41	0.460	3GH1	Predicted nucleotide-binding protein from *Vibrio cholerae*
SS1G_03878	132	-3.79	0.645	1NI3	*Schizosaccharomyces pombe* YchF GTPase
SS1G_04309	177	-4.63	0.461	2QPD	Crystal structure of SusD-like carbohydrate binding protein (YP_001298396.1) from
SS1G_06504	93	-2.38	0.503	4HOI	*Bacteroides vulgatus* ATCC 8482 at 1.70 A resolution
SS1G_07354	129	-3.63	0.589	1UG3	Crystal structure of PAS domain from the mouse EAG1 potassium channel C-terminal portion of human eIF4GI
SS1G_07543	273	-4.27	0.706	2FX5	*Pseudomonas mendocina* lipase
SS1G_08860	141	-3.96	0.526	1XLY	X-ray structure of the RNA-binding protein SHE2p
SS1G_09512	164	-3.65	0.546	3LE0	Lectin Domain of Lectinolysin complexed with Glycerol
SS1G_10915	136	-4.12	0.512	3M1C	Crystal structure of the conserved herpesvirus fusion regulator complex gH-gL
SS1G_11461	114	-3.2	0.526	2 HP3	Penicillin-binding protein 2b (PBP-2b) from *Streptococcus pneumoniae* (strain 5204)
SS1G_12769	88	-2.32	0.585	3S63	Saposin-like protein Na-SLP-1
SS1G_13016	124	-2.76	0.555	3APO	ERAD pathway mediated by the ER-resident protein disulfide reductase ERdj5
SS1G_13142	131	-4.25	0.506	1DCU	Oxidized pea fructose-1,6-bisphosphate phosphatase
SS1G_13235	114	-3.55	0.585	3D3M	C-terminal region of Death Associated Protein 5(DAP5)
SS1G_14000	159	-4.43	0.578	2C9K	Structure of the functional form of the mosquito larvicidal Cry4Aa toxin from *Bacillus thuringiensis* at a 2.8-angstrom resolution

C-score is a confidence score for estimating the quality of models predicted by I-TASSER ranging from -5 (low confidence) to 2 (high confidence). TM score is a quality score for the superimposition of 3D models calculated with TM-align with values in [0;1]. A TM-score >0.5 generally corresponds to the same fold in SCOP/CATH.

### *S. sclerotiorum* effector candidate genes show diverse patterns of expression ***in planta***

**Figure 7:**
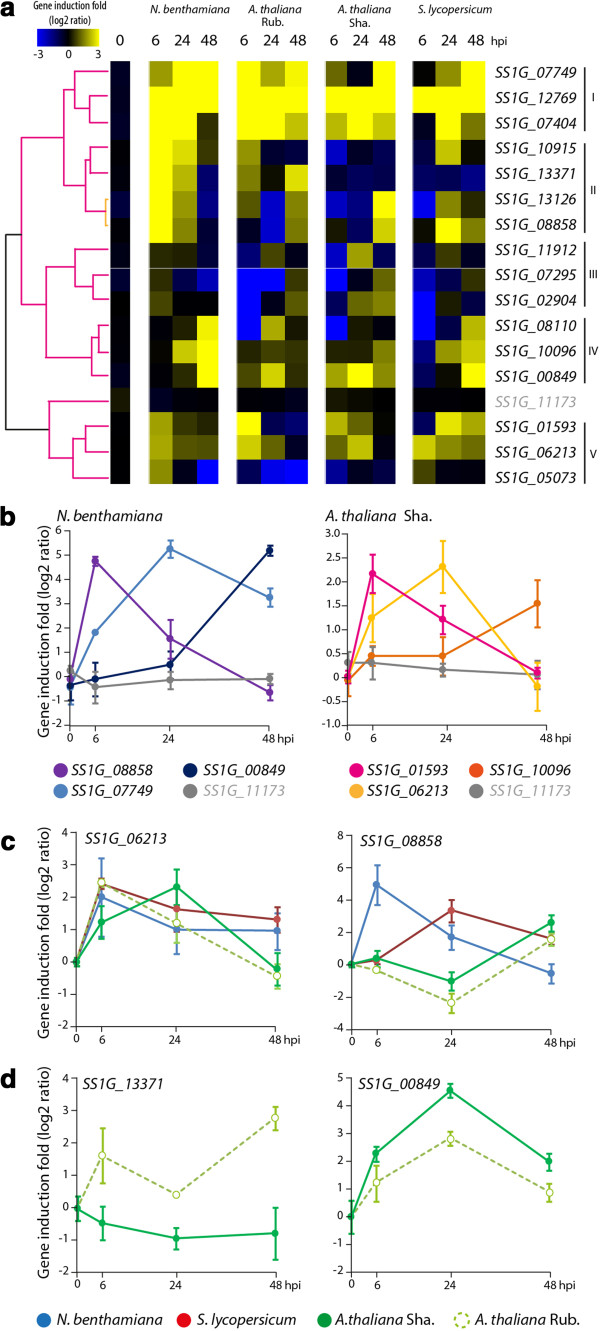
***In planta*** **expression analysis for selected** ***S*** . ***sclerotiorum*** **effector candidates on four different hosts. (a)** Transcriptional profiles of 16 *S. sclerotiorum* effector candidate genes. Overrepresented (yellow) and underrepresented transcripts (blue) *in planta* are shown as log2-fold changes relative to expression *in vitro*, normalized using Actin expression. Hierarchical clustering based on Pearson correlation coefficients delimited five clusters. The *SS1G*_*11173* ubiquitin 16 gene was used as a non-induced control. **(b)** Sequential transcriptional activation of effector gene candidates during the infection of *N. benthamiana* (left) and *A. thaliana* Sha. accession (right). **(c)** *In planta* expression pattern of candidate effector genes showing host-independent expression (*SS1G_06213*, left) and host-dependent expression (*SS1G_08858*, right). **(d)** Differential expression patterns of two candidate effector genes on susceptible (Sha.) and resistant (Rub.) *A. thaliana* accessions. Relative gene expression shown as log2-fold changes relative to expression *in vitro*, normalized using Actin expression. Error bars show standard deviation calculated from two technical replicates on each of three independent biological experiments. Rub., Rubezhnoe; Sha., Shahdara.

To test whether ECs identified in this work could play a role during interaction with host plants, we monitored the expression pattern after plant inoculation by quantitative RT-PCR for 16 EC genes, representative of the four lists of ECs identified through our bioinformatics analyses. For this, we inoculated four different host and model plants, including Tomato, *Nicotiana benthamiana*, *Arabidopsis thaliana* resistant accession Rubezhnoe and susceptible accession Shahdara, and harvested samples at 6, 24 and 48 hours post-inoculation (hpi). *S. sclerotiorum* mycelium grown *in vitro* was used as a reference. We included the *S. sclerotiorum* ubiquitin-conjugating enzyme gene *SS1G_11173* as non-induced control gene and the cerato-platanin gene *SS1G_10096* as an *in planta*-induced control [14]. Eight effector candidate genes showed *in planta* induction >2 on all four host plants tested. One gene (*SS1G_07295*) did not show *in planta* induction ≥2 fold on any plant. Using hierarchical clustering, we grouped effector candidate genes into five clusters according to their expression pattern (Figure 7a). Genes in cluster I show strong induction *in planta* over at least two time points; genes in cluster II showed strong and early induction in *N. benthamiana*, but only moderate and late induction on other host plants; genes in cluster III showed stronger induction on *A. thaliana* Shahdara accession; genes in cluster IV showed late (48 hpi) induction, stronger on *N. benthamiana* and tomato; and genes in cluster V showed moderate induction at early time points (≤24 hpi). The cerato-platanin gene *SS1G_10096* grouped in cluster IV.

The set of 16 ECs analyzed presented diverse expression patterns. We observed peaks of expression on *N. benthamiana* at 6, 24 or 48 hpi for *SS1G_08858*, *SS1G_07749* and *SS1G_00849* respectively illustrating the diversity of induction kinetics. On *A. thaliana* Shahdara accession, peaks of expression occurred at 6, 24 or 48 hpi for *SS1G_01593*, *SS1G_06213* and *SS1G_10096* respectively (Figure 7b). At 24 hpi, extensive cell death was visible on leaves of *A. thaliana* accession Shahdara whereas only limited cell death symptoms were visible on *N. benthamiana* (Additional file 2: Figure S1), suggesting that the activation of host cell death is not the only determinant of *S. sclerotiorum* EC induction. We observed a consistent 2- to 4- fold induction between 6 and 24 hpi for *SS1G_06213* on all four host plants tested. By contrast, *SS1G_08858* was induced >4-fold at 6 hpi on *N. benthamiana*, at 24 hpi on tomato, and at 48 hpi on *A. thaliana* (Figure 7c). This result suggests that *S. sclerotiorum* possess effector genes that are regulated independently of the host being colonized and others that are differentially regulated in a host-dependent manner. Furthermore, *SS1G_13371* was induced >2 fold during infection of *A. thaliana* resistant accession Rubezhnoe, but not during infection of the susceptible accession Shahdara. Conversely, *SS1G_00849* was induced >8 fold during infection of the susceptible accession Shahdara, but only ~4 fold during infection of the resistant accession Rubezhnoe (Figure 7d). This data points towards a versatile repertoire of effector candidates the expression of which can be modulated according to the nature of the host plant being colonized.

## Discussion

In their global analysis of *S. sclerotiorum* genome, Amselem *et al.* [12] identified 603 genes encoding non-CAZYme, non-peptidase secreted proteins. These secretome genes did not appear significantly enriched in genes induced *in planta*. In this study, we combined multiple bioinformatics approaches to identify a total of 745 predicted secreted proteins, among which 486 with experimental evidence for expression *in planta* (SPEPs). The predicted SPEPs include SsNEP1 and SsITL1 that have proposed to be *S. sclerotiorum* virulence factors [8, 44]. Since we have chosen to focus the search for effector candidates on these 486 SPEP genes, we have deliberately ignored genes expressed *in planta* for which experimental evidence is lacking, and enzymes that contribute to the biosynthesis of secondary metabolites as virulence determinants. It is therefore expected that the diversity of *S. sclerotiorum* virulence factors exceeds that of the candidate effectors presented here. Sequence similarity to known fungal effectors is a powerful method to uncover effector families conserved across species [16], that allowed us to identify *S. sclerotiorum* homologs of *B. cinerea* NEP1 [44] and Spl1 [14], *M. oryzae* MGG_15370 and *C. hingginsianum* CHEC91 and CHEC80. To complement this approach, we used PFAM domain and NLS motif searches to reveal additional effector candidates. We identified putative chitin-binding proteins, putative protease inhibitors, cystein-rich proteins and putative nuclear localized proteins. Effectors with chitin binding activity such as *C. fulvum* Ecp6, *M. oryzae* Slp1 and *M. graminicola* Mg3LysM function in suppressing plant immunity [18, 20, 59]. Similarly, SsITL1 (SS1G_14133) integrin-like secreted protein suppresses plant jasmonic acid and ethylene signaling pathways and enhances susceptibility [8]. These findings suggest that *S. sclerotiorum* secretes proteins able to suppress plant immunity.

The comparative analysis of *Fusarium graminearum* secretome and genomes of other ascomycetes revealed a high level of conservation with only 25 *F. graminearum* specific out of 574 secreted proteins [60]. The taxonomic distribution of *S. sclerotiorum* SPEP homologues analyzed in this work supports the conservation of more than 50% of SPEP genes across ascomycetes. As proposed by Brown *et al*. [60], these core SPEP genes may support *S. sclerotiorum* epiphytic growth and highlight important distinctions between multiple phases in infection by this fungus [61]. Nevertheless it also revealed 70 SPEP genes (14%) specific to *S. sclerotiorum*, many of which are unannotated proteins. The systematic prediction of their 3D structure allowed identifying putative structural analogs of some predicted SPEPs and suggests that they may carry unique functions to assist *S. sclerotiorum* pathogenicity. It will be interesting to take advantage of these predictions to test the biological function of these effector candidates and confront them to experimentally determined structures. Furthermore, in spite of the limited sequence diversity included in the dataset analyzed here, we were able to detect signatures of positive selection in five *S. sclerotiorum* SPEP genes (2.5% of genes analyzed). Similar frequency (3.2%, 21 out of 642 genes) has been reported in *B. cinerea* [35]. In the future, an in depth exploration of sequence diversity in *S. sclerotiorum* should allow to reveal more sites subjected to selection. SPEP genes for which positive selection has been detected encode cell wall degrading enzymes, including *SS1G*_*07749* encoding a putative xylanase. This protein is related to *B. cinerea* Xyn11 considered as a Pathogen Associated Molecular Pattern (PAMP) [9]. The detection of positive selection in *SS1G*_*07749* is therefore consistent with the hypothesis that PAMPs may be characterized by signatures of positive selection in a background of strong negative selection [50]. It may therefore be hypothesized that a subset of *S. sclerotiorum* critical secreted enzymes are engaged in an evolutionary arms race with plant pattern recognition receptors, driving opposing forces of natural selection *S. sclerotiorum* effector genes. Since plant inhibitors are known for many fungal cell wall degrading enzymes, it is also possible that an evolutionary arms race with plant inhibitors drives the evolution of some *S. sclerotiorum* effector candidates [53, 62, 63]. Remarkably, we also identified 14.4% of species-specific SPEP genes. The extent to which evolutionary constraints imposed by a broad host range contributes to diversification in the effector candidate repertoire of *S. sclerotiorum* remains to be determined. Detailed functional analysis of effector gene alleles and their plant targets will be needed to address this question.

Although *S. sclerotiorum* is considered as a typical necrotroph, there is evidence that it colonizes plant tissues through multiple phases involving important transcriptional and physiological reprogramming [61]. Consistent with this model, the phytotoxin oxalic acid dampens plant immune responses at the initial stages of infection and later enhances programmed cell death [7]. In this work, we report the sequential transcriptional activation of *S. sclerotiorum* candidate effector genes. A >2 fold induction was measured at 6 hpi for several effector candidate genes, whereas no necrotic symptoms are visible at this time, except on *A. thaliana* Shahdara accession. This suggests that the sequential secretion of effectors is required for the efficient induction of host cell death by *S. sclerotiorum*, or that some secreted proteins could contribute to *S. sclerotiorum* virulence independently of host cell death activation. By comparing the expression pattern of selected *Blumeria graminis* f. sp. *hordei* genes grown on barley and on *A. thaliana*, Hacquard *et al*. [64] concluded that very divergent hosts do not significantly alter the fungal gene expression program. The expression pattern of some *S. sclerotiorum* ECs is indeed independent on the host plant being colonized (e.g. *SS1G*_*06213*). Nevertheless, other ECs showed differential regulation in a host-dependent manner (e.g. *SS1G*_*08858*, *SS1G*_*13371*). The transcriptional activation of distinct set of effectors depending on the host being colonized has also been reported for the generalist root endophyte *Piriformospora indica* in barley and *A. thaliana* [65]. The growing number of transcriptomic studies on various pathosystems should help determine whether host-dependent modulation of effector gene expression differs according to pathogens lifestyle or host range. We speculate that the white mold fungus benefits from a versatile repertoire of secreted proteins with diverse functions, evolution and expression patterns, to successfully infect a wide range of host plants. A systematic characterization of *S. sclerotiorum* transcriptome on multiple hosts and the functional analysis of differentially regulated effector genes should prove useful to decipher the molecular determinants of quantitative disease resistance and host range in this fungal pathogen.

## Conclusions

In this work, we explored systematically the diversity of candidate virulence genes in the necrotrophic fungal pathogen *S. sclerotiorum* using *in silico* structure and evolution analyses. We report the identification of 486 *S. sclerotiorum* secreted proteins expressed *in planta*, including 78 ECs. We have analyzed *in planta* expression for a representative subset of 16 ECs, highlighting diverse predicted functions and expression patterns. This study reveals that besides plant degrading enzymes, *S. sclerotiorum* genome encodes numerous predicted secreted proteins that may be involved in the interaction between the fungus and its host plants. It will facilitate future investigation on their relevance in the infection process and sheds new light on the underestimated complexity of host colonization by necrotrophic plant pathogens.

## Methods

### Secretome prediction and annotation

We used complete genome and predicted proteomes of *Sclerotinia sclerotiorum* strain 1980 v.2, *Botrytis cinerea* strain b05.10 v.1 and strain t4 v.1 described in [12]. The presence of secretion signals was predicted with SignalP v.2 and v.4 [66, 67], transmembrane helices and GPI anchor sequence were predicted with TMHMM [68] and GPIsom [69] respectively. For the identification of genes expressed *in planta*, microarrays data for gene induction fold at 2 days post inoculation on sunflower cotyledons and Expressed Sequence Tags (ESTs) from [12] were used. ESTs were assigned to the *S. sclerotiorum* predicted transcript giving the lowest e-value in a BLASTN search. Genes were considered expressed *in planta* when either (i) showing induction fold ≥1 in during sunflower infection in microarrays data or (ii) being assigned at least one EST in either infection cushion, infected *B. napus* or infected tomato library. *S. sclerotiorum* predicted proteins were annotated using Blast2GO [70], PFAM [71] and NLStradamus [72]. Predicted proteins shorter than 40 amino-acids were excluded from the analysis. PFAM domains were annotated using HMMER3 searches against the PFAM 26.0 database [71]. We defined non-annotated predicted SPEPs as having no hit to PFAM_A with e-value <0.1. For the identification proteins similar to known fungal effectors, BlastP searches against a local database of 191 effectors with an e-value cutoff of 1e^-3^.

### Definition of ortholog clusters and natural selection analysis

Core ortholog groups (COGs) between *S. sclerotiorum* 1980 and *B. cinerea* b05.10 or *B. cinerea* t4 proteomes were identified using Inparanoid7 [73] with the following parameters: score cutoff 40 bits; sequence overlap cutoff 0.5; group merging cutoff 0.5; scoring matrix BLOSUM62. COGs in which a length difference >10 amino-acids existed between *S. sclerotiorum* and *B. cinerea* were discarded. Pairwise ortholog alignments were generated using the needleall program from the EMBOSS package using the following parameters: gapopen 50.0; gapextend 0.2; minscore 100.0; aformat3 MARKX3. Needleall output files were parsed into .axt alignments used as input in Ka/Ks calculator2 [74]. Ka/Ks ratios were calculated for all COG pairs using Yn00 method [28]. The identification of codon sites under positive selection was achieved through Bayesian inference using the Selecton2.2 server [75] with the “Positive selection enabled (M8, beta + w > =1)” evolutionary model with 8 categories, on alignments of *S. sclerotiorum* 1980, *B. cinerea* b05.10 and *B. cinerea* t4 orthologs.

### Protein structure modeling and analysis

Protein structure modeling was performed with the I-TASSER server [76] and rendered using UCSF Chimera [77]. Site-specific alignment consensus and Ka/Ks ratios were mapped onto protein models using the ‘define attribute’ function in UCSF Chimera. Moving average over a 3 amino-acid window of the percentage consensus in a 99 homologous protein alignment was used to characterize conservation in SS1G_01593 family. Structural analogs were identified using the TM-align program in I-TASSER.

### Taxonomic distribution and phylogenetic analyses

Fungal taxonomy trees are based on [16]. The presence of SPEP homologs in 234 fungal species was assessed using BlastP searches against JGI fungi Gene Catalog Proteins [78] with an e-value cutoff of 1e^-5^ without low complexity filter. Among retrieved homologs, proteins that had no signal peptide detected with SignalP4 or SignalP2 were discarded. For the global analysis of taxonomic distribution of SPEP genes, the predicted proteomes of *Neurospora crassa*, *Magnaporthe oryzae*, *Verticilium dahlia*, *Fusarium oxysporum*, *Stagonospora nodorum*, *Pyrenophora tritici*-*repentis*, *Alternaria brassicicola*, *Leptosphaeria maculans*, *Mycosphaerella graminicola*, *Aspergillus flavus*, *Cryptococcus neoformans* and *Rhizopus oryzae* were used in local BlastP searches with e-value cutoff 1. For each SPEP gene, BlastP scores for all hits in a given species were summed up, and SPEP genes were considered as absent if total score is <2. Phylogenetic trees for SS1G_13371 family was generated using the parsimony method with 100 bootstrap replicates with the Extended Majority rule, as implemented in the protpars and consense programs of the Phylip 3.67 package [79].

### Sequence-based clustering and genome distribution of duplicated genes

*S. sclerotiorum* 1980 and *B. cinerea* b05.10 proteins were clustered based on sequence similarity by Markov clustering using the orthoMCL function in Biolayout 3D [80]. A self BlastP search on the combined *S. sclerotiorum* 1980 and *B. cinerea* b05.10 complete proteomes with e-value cutoff 1e^-30^ was used as input for orthoMCL. Repeats and transposable elements were identified using RepeatMasker on *S. sclerotiorum* 1980 supercontigs with the cross_match method at slow speed and “Fungi” as a DNA source. Genomic distances and genome architecture heatmaps were generated according to [81].

### Plant and fungus cultivation, inoculation procedure

*Arabidopsis thaliana* accession Shahdara and Rubezhnoe-1 were grown in Jiffy pots for four weeks at 22°C with cycles of 9 hours of light per 24 hours. Tomato (*Solanum lycopersicum* cv. Heinz) were grown for six weeks in pots containing disinfected soil in a greenhouse at 23°C with cycles of ~14 hours of light per 24 hours. *Nicotiana benthamiana* plants were grown for four weeks at 21°C with cycles of 16 hours of light per 24 hours. *S. sclerotiorum* strain S55 was first grown for 4 days on PDA plates at 25°C in the dark. Fifty mL of liquid PDB medium were inoculated with 3 agar plugs of PDA cultures and incubated for 4 days at 25°C in the dark, with 150 rpm shaking. Three independent inoculation experiments were performed in which fully grown plant leaves were cut and placed right side up on a wet paper towel in large petri dishes. Mycelium was washed twice in PDB, filtered on Miracloth (Calbiochem, CA), and spread over whole leaf surfaces. Inoculated leaves were incubated for up to 3 days at 25°C with 14 hours of light per 24 hours.

### Effector candidate gene expression by quantitative RT-PCR

Plant leaves were harvested immediately and 6, 24 and 48 hours after inoculation, and ground in liquid nitrogen. Total RNA was extracted using a Nucleospin RNA II kit (Machery- Nagel) according to manufacturer’s instructions. RNAs were analyzed and quantified on an Agilent 2100 Bioanalyzer. The first-strand cDNA was synthesized using TRT reverse transcriptase (Roche) according to manufacturer’s instructions. Real-time PCR reactions included 3.5 μL of SYBR green mix (Roche), 1 μL of 5 μM primers (Additional file 3: Table S2) and 200 ng of cDNA. Reactions were performed on a Light Cycler 480 II machine (Roche) under the following conditions: 95°C for 5 minutes; 45 cycles of 95°C for 15 seconds, 65°C for 20 seconds and 72°C for 20 seconds; then 95°C for 10 seconds; 65°C for 15 seconds followed by a progressive in increase in temperature at 0.11°C/second up to 95°C to obtain melt curve. *S. sclerotiorum* actin (*SS1G*_*08733*) and ubiquitin 16 (*SS1G*_*11173*) genes were used as controls. The expression of effector gene candidates relative to Ct values of the control genes was determined and analyzed using the LightCycler 480 SW 1.5 software. Fungal cultures were grown *in vitro* for 3 days and either harvested immediately (Day 0) or inoculated to plants. Values are given as log2 ratio over Day 0 expression. Error bars represent standard deviation calculated from two technical replicates on each of three independent biological experiments.

## Electronic supplementary material

Additional file 1: Table S1: Summary table of annotations for the 486 SPEP genes, including homology, domain and motif searches and expression data. Inclusion into sequence-based clusters, into COG groups, Ka/Ks values and closest structural analogs are indicated where relevant. (XLSX 313 KB)

Additional file 2: Figure S1: Representative symptoms of detached leaves inoculated by *S. sclerotiorum* S55 at 6, 24 and 48 hours post inoculation (hpi). (PDF 68 KB)

Additional file 3: Table S2: List of primers used in this work. (XLSX 8 KB)
