# Supplementary material for: Secretome analysis reveals effector candidates associated with broad host range necrotrophy in the fungal plant pathogen Sclerotinia sclerotiorum
Source: BMC Genomics. 2014 May 4;15(1):336. doi: 10.1186/1471-2164-15-336 (PMC4039746; doi:10.1186/1471-2164-15-336)
Supplement: Supplementary file 2 — Additional file 2: Figure S1: Representative symptoms of detached leaves inoculated by S. sclerotiorum S55 at 6, 24 and 48 hours post inoculation (hpi). (PDF 68 KB) [file 12864_2014_6074_MOESM2_ESM.pdf]

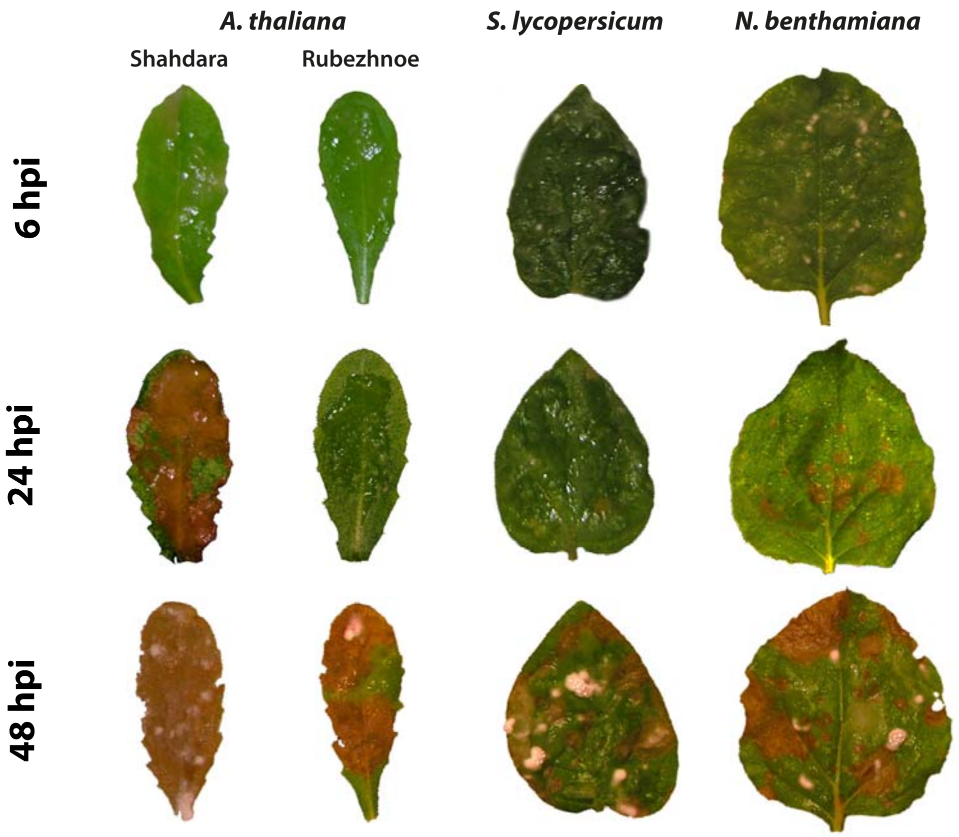

Figure S1. Representative symptoms of detached leaves inoculated by *S. sclerotiorum* S55 at 6, 24 and 48 hours post inoculation (hpi).
